# Awareness, Knowledge, Attitudes, and Practices of Diabetic Nephropathy Among the General Population in Hafr Al Batin, Saudi Arabia: A Cross-Sectional Study

**DOI:** 10.7759/cureus.71987

**Published:** 2024-10-21

**Authors:** Ashafq Ahmad, Abdulaziz A Alshammari, Abdullah M Aldhafeeri, Abdullah L Alharbi, Othman H Aldhafeeri, Thoini H Aldhafeere, Majed M Aldahmashi, Ali H Aldhafeeri, Adel A Alharbi, Esraa M Haji

**Affiliations:** 1 Department of Pharmacy Practice, College of Pharmacy, University of Hafr Al Batin, Hafr Al Batin, SAU; 2 College of Pharmacy, King Faisal University, Al-Ahsa, SAU; 3 Department of Therapeutics, Qassim Health Cluster, Buraydah, SAU; 4 Department of Pharmaceutical Chemistry, College of Pharmacy, University of Hafr Al Batin, Hafr Al Batin, SAU

**Keywords:** attitude, awareness, cross-sectional, diabetic nephropathy, knowledge, practice, public health, saudi arabia

## Abstract

Background

Diabetic nephropathy, a leading cause of end-stage renal disease (ESRD), plays a part in the critical healthcare burden globally, especially in Saudi Arabia, where the prevalence of diabetes cases is among the highest worldwide. This study aims to assess the awareness, knowledge, attitudes, and practices (A-KAP) in relation to diabetic nephropathy among the general population in Hafr Al Batin, Saudi Arabia.

Methods

A cross-sectional study was conducted between January and September 2024, involving 406 participants with a minimum age range of 18 years and older. Data were collected using a self-administered questionnaire, disseminated through social media platforms, i.e., WhatsApp and Facebook, and analyzed using IBM SPSS Statistics for Windows, Version 28.0 (Released 2021; IBM Corp., Armonk, New York, United States). The chi-squared test, Fisher's exact test, and logistic regression analyses were performed to examine the associations between demographic variables and A-KAP scores, with a p≤0.05.

Results

Of the participants, 53.7% were male, and the largest age group was 20-29 years old (46.8%). The results revealed significant associations between knowledge scores and gender (p=0.033), age (p=0.012), and BMI (p=0.002). Females and participants aged 40-49 years had higher knowledge scores. Furthermore, 58.4% of participants were unaware of their BMI, and those with higher BMI scores showed lower knowledge levels. Regarding attitudes, older participants (aged 40 and above) have demonstrated more positive attitudes toward diabetic nephropathy (p=0.02). Practices related to nephropathy screening were deficient, with 84.2% of participants having never been screened and 68.7% never checking their kidney function. A significant proportion of participants, specifically 71.2%, indicated a lack of regular engagement in physical exercise, highlighting a notable prevalence of physical inactivity.

Conclusion

This study reveals notable differences in awareness, perceptions, and behaviors related to diabetic nephropathy among the general population in Hafr Al Batin, Saudi Arabia. Females, individuals aged 40-49 years, and participants with lower BMI exhibited higher knowledge scores, while health maintenance practices such as nephropathy screening and kidney function checks were predominantly inadequate. Public health initiatives ought to prioritize enhancing awareness, advocating for regular screening, and fostering physical activity to reduce the risk of diabetic nephropathy. Future research should focus on identifying and overcoming the obstacles to healthcare access and preventive services for this population.

## Introduction

Diabetic nephropathy, a leading cause of end-stage renal disease (ESRD), represents a critical health burden globally, particularly in regions with a high prevalence of diabetes mellitus [1]. It is a microvascular complication associated with diabetes, which advances insidiously and may result in permanent kidney damage. This condition considerably detracts from quality of life and amplifies healthcare expenditures [2]. Saudi Arabia is recognized as one of the countries with the highest prevalence of diabetes and its related complications. Approximately one-third of the Saudi population with type 2 diabetes suffers from diabetic nephropathy [3]. The highest comparative diabetes prevalence rates in 2021 are reported in Pakistan (30.8%), French Polynesia (25.2%), and Kuwait (24.9%) [4]. It is predicted that 42.5% of ESRD cases in Saudi Arabia are attributable to diabetes [5]. In Saudi Arabia, diabetes poses a significant public health challenge, with prevalence rates that are among the highest globally. This alarming trend underscores the urgent need to address the related complications, particularly diabetic nephropathy [6].

Public awareness, knowledge, and preventive practices are crucial in mitigating the onset and progression of diabetic nephropathy [7]. Gaining insights into the general population's awareness, knowledge, attitudes, and practices (A-KAP) pertaining to this condition is crucial for the development of effective educational campaigns and preventative strategies [8]. The International Diabetes Federation (IDF) Diabetes Atlas estimated that in 2017, the prevalence of diabetes was 10.9%, and it estimated that there were 114 million people living with diabetes and 61 million people with undiagnosed diabetes [4]. In the United States, up to 19% of people with type 2 diabetes have overt nephropathy [9]. However, there is limited data on the A-KAP of diabetic nephropathy in Saudi Arabia, particularly in regions like Hafr Al Batin, where the population may have unique sociodemographic and healthcare access challenges.

This research seeks to address a crucial knowledge deficit by evaluating the levels of awareness, attitudes, practices, and understanding of diabetic nephropathy among the general population in Hafr Al Batin. By pinpointing deficiencies in both awareness and behavioral practices, this study aims to offer evidence-based recommendations that can inform public health initiatives, thereby improving early detection and preventive measures for diabetic nephropathy in Saudi Arabia.

## Materials and methods

Study design

A cross-sectional study was performed among the general population in Hafr Al Batin, Saudi Arabia. Data were obtained by community-based, self-administered questionnaires from January to September 2024. The questionnaire was validated and checked for reliability as well. The research adhered to the Declaration of Helsinki, and ethical approval was secured by the Ethical Committee of the University of Hafr Al Batin, Hafr Al Batin, Saudi Arabia (approval number: HPO-05-FT-24-06).

Inclusion and exclusion criteria

Individuals aged 18 years and older were enrolled in the study. The subjects have a basic education degree.

Sample size

The sample size for this investigation was calculated using the Raosoft software (Raosoft Inc., Seattle, Washington, United States), employing the single proportion sample size calculation. Our objective was to achieve a precision of 95% CI which corresponds to a significance level of 0.05. [10].

Data collection tool

The questionnaire (see Appendices) was modified from prior studies in English and subsequently translated into Arabic [11-13]. The Arabic version was translated by two independent Arabic-English translators unaware of the original English version's existence for back-translation purposes [14]. The content validity was assessed in the initial 20 participants using the following three questions: (1) "Do you find the questions pertinent to the subject matter?", (2) "Is the questionnaire challenging to complete?", and (3) "Specify the questions you wish to modify or include, and in what manner?". Minor adjustments to the layout were implemented in response to comments. The questionnaire's reliability was assessed using the computation of Cronbach's alpha coefficient. A reliability index of at least 0.6 was deemed acceptable [15].

The demographic variables included in the study were gender, age, marital status, educational level, occupation, and comorbidities. It also included questions to assess the participant's knowledge, attitudes, and practices related to diabetic nephropathy, their choice of a healthcare professional, and treatment options for diabetic nephropathy. For the knowledge, attitudes, and practice sections, a five-point Likert scale with responses ranging from strongly agree to strongly disagree was recorded.

Subjects were instructed to complete the self-administered questionnaires. The survey was disseminated via social media platforms, i.e., WhatsApp and Facebook.

Statistical analysis

The analysis was done using IBM SPSS Statistics for Windows, Version 28.0 (Released 2021; IBM Corp., Armonk, New York, United States). Categorical data were represented as frequencies and percentages. Continuous data were represented using medians and interquartile ranges (IQR) or means and standard deviations (SD), as specified. The chi-squared and Fisher's exact tests were employed to compare the variables, with a p-value of less than 0.05 being statistically significant for all analyses. The association between knowledge, attitudes, and practice was measured using logistic regression (univariate and multivariate).

## Results

Participant characteristics

Table 1 presents the demographic and clinical characteristics of the study participants in descending order for each factor. For gender, 226 (53.7%) were male, followed by 180 (44.3%) females. Regarding age groups, 190 (46.8%) were aged 20-29 years, 86 (21.2%) were aged 30-39 years, 82 (20.2%) were aged 40-49 years, 35 (8.6%) were aged 10-19 years, and 13 (3.2%) were aged 50 and above. Regarding BMI, 237 (58.4%) did not know their BMI, followed by 454 (13.4%) with a BMI greater than 29 kg/m² and 50 (12.3%) with a BMI of 18.5-24.9 kg/m². In terms of educational attainment, the largest group of participants had a bachelor's degree, representing 222 (54.7%), followed by 90 (22.2%) who had completed high school and 68 (16.7%) with a diploma. Only 15 (3.7%) had a master's or PhD degree, and 11 (2.7%) had completed secondary school. Regarding occupation, 176 (43.3%) of respondents were employed, while 142 (35%) were students, and 88 (21.7%) were unemployed. For marital status, 204 (50.2%) were married, 191 (47.1%) were single, eight (1.9%) were divorced, and three (0.7%) were widowed.

**Table 1:** Demographic characteristics of participants (n=406) HTN: hypertension

Variables	N	%
Gender		
Female	180	44
Male	226	54
Age groups (in years)		
10-19	35	9
20-29	190	47
30-39	86	21
40-49	82	20
50 and above	13	3
BMI (kg/m^2^)		
<18.5	19	5
18.5-24.9	50	12
25-29	46	11
>29	54	13
I don't know	237	58
Education		
Secondary school	11	3
High school	90	22
Diploma	68	17
Bachelors	222	55
Masters/PhD	15	4
Occupation		
Student	142	35
Employed	176	43
Unemployed	88	22
Marital status		
Single	191	47
Married	204	50
Divorced	8	2
Widowed	3	1
Comorbidities		
Healthy (no disease)	318	78
Kidney failure	1	1
HTN, kidney failure	1	1
HTN, diabetes, lipid disorder	2	1
Diabetes, lipid disorder	3	1
HTN, lipid disorder	5	1
HTN, diabetes	6	1
Lipid disorder	12	3
HTN	13	3
Diabetes	23	6
Others	22	5

In terms of comorbidities, the majority of participants were healthy, with 318 (78.3%) reporting no disease. Among those with health conditions, the most common was diabetes, reported by 23 (5.6%) participants, followed by hypertension at 13 (3.2%) and lipid disorder at 12 (2.9%).

Awareness regarding diabetic nephropathy

**Figure 1:**
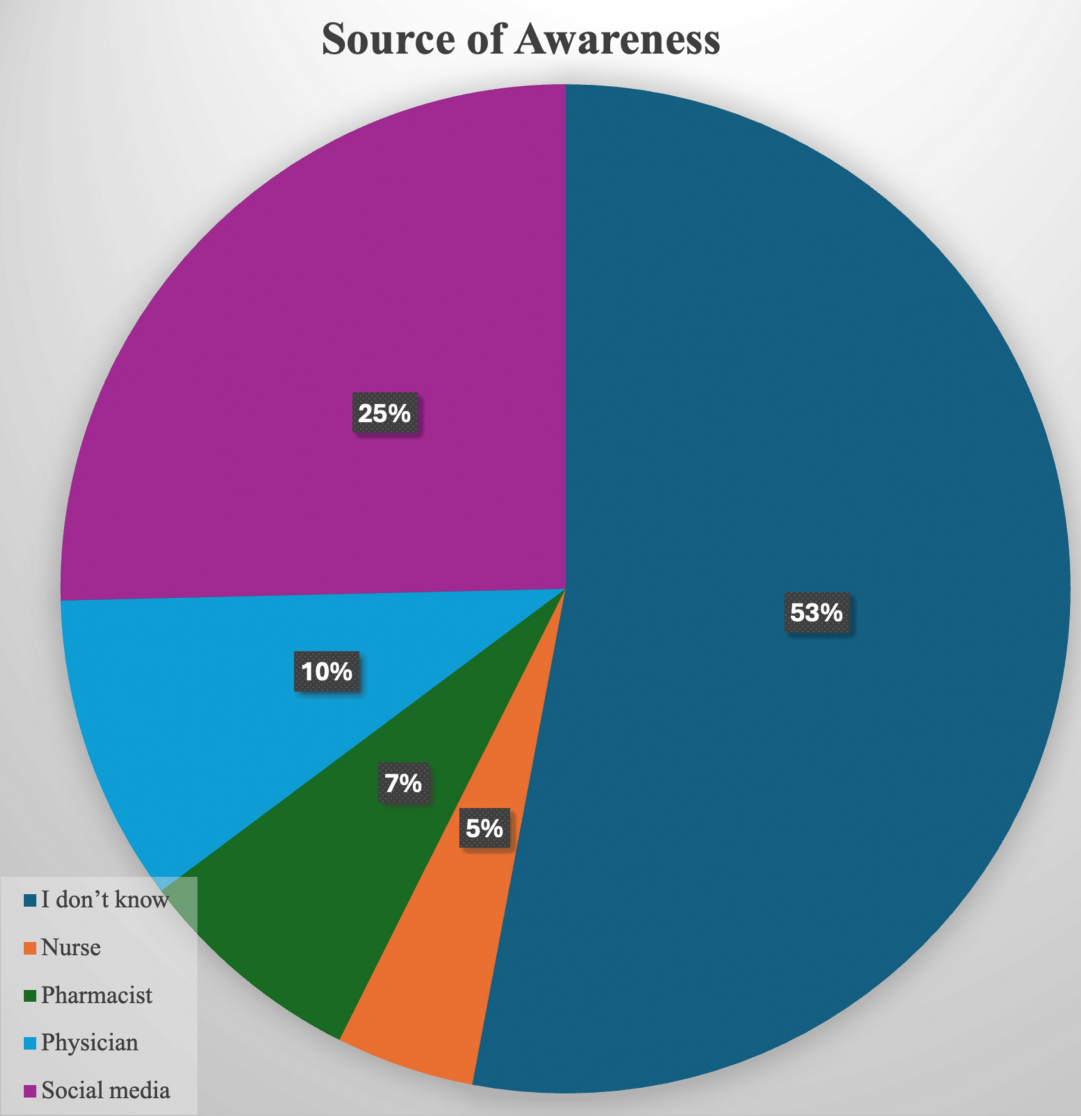
Source of information regarding diabetic nephropathy

The pie chart in Figure 1 depicts the many sources of awareness of diabetic nephropathy within the examined population. The predominant group, comprising 53%, pertains to persons who answered "I don't know," signifying a substantial fraction of the population is uninformed or unable to identify a source of information regarding diabetic nephropathy.

Social media constitutes 25% and is the second most prevalent source of awareness, underscoring the significance of digital platforms in the distribution of health information. Ten percent of respondents identify physicians as their source of awareness, indicating that healthcare providers have a significant yet not predominant role in public education.

Pharmacists constitute 5%, while nurses represent 7%, indicating minimal contributions from other healthcare professions in promoting awareness.

Frequency of renal function test

Table 2 presents the frequency of kidney function tests among the study participants. The majority, 276 (68%), reported that they have never had a kidney function test. In contrast, 97 (23.9%) indicated that they undergo kidney function tests annually. A smaller proportion of participants reported having kidney function tests more frequently, with 17 (4.2%) doing so every six months and 16 (3.9%) every three months.

**Table 2:** Frequency of kidney function test

Kidney function test	N	%
Never	276	68
Three monthly	16	4
Six monthly	17	4
Annually	97	24

Practice regarding diabetic nephropathy

Table 3 presents the participants' responses regarding various health practices. Regarding diabetic nephropathy screening, 342 (84.2%) of participants reported that they had never been screened, while only 64 (15.8%) had been screened. In terms of familiarity with diabetic nephropathy, 128 (31.5%) indicated that a family member or friend had experienced it, while 278 (68.5%) had no such experience.

**Table 3:** Participants' practices regarding diabetic nephropathy

Practices	Groups	N	%
P1. Have you ever been screened for diabetic nephropathy?	No	342	84
	Yes	64	16
P2. Have any of your family members or friends experienced diabetic nephropathy?	No	278	69
	Yes	128	32
P3. Have you ever had your kidney function checked?	No	279	69
	Yes	127	31
P4. Do you exercise regularly?	No	289	71
	Yes	117	29
P5. How often do you check your kidneys?	Never	276	68
	Quarterly	16	4
	Biennially	17	4
	Annually	97	24
P6. How many medications do you take?	No medication	300	74
	One	56	14
	Two	26	6
	Three	6	2
	More than three	18	4
P7. How often do you check your blood sugar?	Never	233	57
	Quarterly	39	10
	Biennially	41	10
	Annually	93	23
P8. What is your daily water intake?	I don't remember	72	18
	4 cups	125	31
	6 cups	95	23
	8 cups	81	20
	10 cups	33	8

For kidney function checks, 279 (68.7%) of participants reported never having their kidney function checked, whereas 127 (31.3%) had done so. Regarding exercise, 289 (71.2%) of participants reported that they do not exercise regularly, while 117 (28.8%) said they do. Kidney check frequency showed that 276 (68%) never check their kidneys and 97 (23.9%) do so annually, 17 (4.2%) every two years, and 16 (3.9%) quarterly.

When asked about medication use, the majority, 300 (73.9%), reported taking no medications, while 56 (13.8%) take one, 26 (6.4%) take two, 6 (1.5%) take three, and 18 (4.4%) take more than three medications. Regarding blood sugar monitoring, 233 (57.4%) reported that they never check their blood sugar, and 93 (22.9%) do so annually, 41 (10.1%) every two years, and 39 (9.6%) quarterly.

As for daily water intake, 125 (30.8%) of participants drink four cups per day, 95 (23.4%) drink six cups, 81 (20%) drink eight cups, and 33 (8.1%) drink 10 cups, while 72 (17.7%) reported that they do not remember how much water they drank.

Association of demographic characteristics with knowledge score

Table 4 presents the association of demographic characteristics with knowledge scores. The results show a statistically significant difference in mean values between genders, with females (41.27±1.91) having higher scores compared to males (32.82±2.27), as indicated by a p-value of 0.033. Age groups also show a significant difference (p=0.012), with the 40-49 age group having the highest mean (44.59±1.96) and younger age groups having lower means. BMI categories demonstrate a significant difference (p=0.002), with those in the <18.5 and 18.5-24.9 BMI ranges having higher means compared to others. Education shows a trend toward significance (p=0.077), with those holding a master's/PhD having higher means. However, occupation and marital status do not show statistically significant differences, as their p-values are greater than 0.05.

**Table 4:** Association of demographic characteristics with knowledge score

Variables	Mean±SD	P-values
Gender		
Female	41±2	0.033
Male	33±2	
Age groups (in years)		
10-19	31±2	0.012
20-29	41±2	
30-39	32±2	
40-49	45±2	
50 and above	41±2	
BMI (kg/m^2^)		
<18.5	43±2	0.002
18.5-24.9	44±2	
25-29	41±2	
>29	41±2	
I don't know	33±2	
Education		
Secondary school	32±3	0.077
High school	32±2	
Diploma	32±3	
Bachelors	40±2	
Masters/PhD	42±2	
Occupation		
Student	41±2	0.148
Employed	42±2	
Unemployed	39±2	
Marital status		
Single	40±2	0.545
Married	41±2	
Divorced	42±2	
Widowed	41±3	

Association of demographic characteristics with attitude

Table 5 indicates a substantial difference in mean values among age groups, with older persons (namely, those aged 40 and beyond) exhibiting greater mean values than their younger counterparts (p=0.02). This indicates that age may be a significant factor affecting the outcome. The education level exhibits borderline significance (p=0.05), suggesting a potential weak correlation, as people possessing a diploma or secondary education demonstrate marginally elevated mean values relative to those with higher educational attainment. Additional variables, including gender, BMI, occupation, and marital status, exhibit no statistically significant differences, as their p-values were above 0.05, indicating that these factors may lack substantial impact on the outcome of this study.

**Table 5:** Association of demographic characteristics with attitude score

Variables	Mean±SD	P-values
Gender		
Female	32±2	0.31
Male	33±4	
Age groups (in years)		
10-19	34±3	0.02
20-29	33±2	
30-39	33±2	
40-49	34.90±2	
50 and above	35±2	
BMI (kg/m^2^)		
<18.5	35±1	0.22
18.5-24.9	35±3	
25-29	34±2	
>29	34±1	
I don't know	34±2	
Education		
Secondary school	36±2	0.05
High school	35±3	
Diploma	36±2	
Bachelors	34±1	
Masters/PhD	35±2	
Occupation		
Student	34±2	0.18
Employed	34±3	
Unemployed	34±2	
Marital status		
Single	34±2	0.54
Married	34±2	
Divorced	35±2	
Widowed	35±3	

Association of demographic characteristics with practice

The findings reveal a notable disparity between genders, with males exhibiting a higher mean value (8.90±3.98) than females (7.65±2.11), as evidenced by a p-value of 0.02. This indicates that gender could be a significant issue. No statistically significant differences were identified for the other variables since their p-values exceeded 0.05. Age groups, BMI, education, occupation, and marital status exhibit no significant connections with the outcome; nevertheless, some variance in means is observed between categories, notably greater values in those with a BMI exceeding 29 and those possessing diplomas. These results are explained in Table 6.

**Table 6:** Association of demographic characteristics with practice score

Variables	Mean±SD	P-values
Gender		
Female	8±2	0.02
Male	9±4	
Age groups (in years)		
10-19	8±3	0.23
20-29	8±2	
30-39	7±2	
40-49	7±2	
50 and above	7±2	
BMI (kg/m^2^)		
<18.5	8±1	0.21
18.5-24.9	7±3	
25-29	8±2	
>29	10±1	
I don't know	8±2	
Education		
Secondary school	8±2	0.09
High school	7±3	
Diploma	8±2	
Bachelors	8±1	
Masters/PhD	8±2	
Occupation		
Student	7±2	0.19
Employed	7±3	
Unemployed	8±2	
Marital status		
Single	8±2	0.45
Married	8±2	
Divorced	9±2	
Widowed	7±3	

## Discussion

This study offers a comprehensive analysis of the awareness and behavior toward diabetic nephropathy among the general population in Hafr Al Batin, Saudi Arabia. Diabetic nephropathy carries high morbidity and mortality [16]. Our findings revealed varying degrees of knowledge and practice across different demographics, with significant correlations between age, gender, and BMI with the participants' knowledge scores. The female participants of our study exhibited higher knowledge scores as compared to males. Our results also reflect a substantial gap in health practices and screening behaviors, with a significant majority of the population never having been screened for diabetic nephropathy or checked their kidney function. Socioeconomic factors play a significant role, as lack of awareness, financial constraints, and limited access to healthcare facilities can prevent individuals from seeking or obtaining necessary screenings [17]. A study based on the Saudi National Diabetes Registry has found that the prevalence of diabetic nephropathy, including microalbuminuria, macroalbuminuria, and ESRD, is 10.8% [18].

The current study found a significant impact on knowledge scores, where females exhibited higher scores than males (mean±SD: 4.27±1.91 vs. 3.82±2.27, p=0.033), and scores varied significantly across age groups, with the highest knowledge observed among participants aged 40-49 years (mean±SD: 4.59±1.96, p=0.012). In contrast, an Indian study conducted by Bansal et al. found no significant differences in awareness scores between male and female patients or across different residential areas but noted higher awareness in older patients, particularly those aged 50 years and above [19]. Similarly, a study conducted in Palestine reported that higher knowledge scores were significantly associated with younger patients (under 55 years), normal BMI, and higher educational levels, with city residency also playing a role in increased knowledge scores [11]. This reflects a varied trend across different populations.

Additionally, our findings revealed that a concerning 58.4% of participants were unaware of their BMI and the knowledge scores were significantly lower among those unaware of their BMI (3.70±2.10, p=0.002), emphasizing the need for public health initiatives to educate the population on the importance of monitoring body weight. Education and awareness are crucial in preventing diabetic nephropathy. Patients with diabetes should be aware of the risk factors for diabetic nephropathy and the importance of regularly monitoring kidney function [7].

In the current study, 58.4% of participants were unaware of their BMI, and those unaware had significantly lower knowledge scores (3.70±2.10, p=0.002). It has been reported that high BMI is significantly associated with adverse renal events, including diabetic nephropathy, in patients with diabetes [20]. Additionally, Lu et al. found that higher BMI levels were causally linked to an increased risk of diabetic nephropathy and lower estimated glomerular filtration rate (eGFR) levels, particularly in women [21]. Furthermore, it has been reported in a cross-sectional study that there is a strong association between normal BMI and better knowledge scores regarding the prevention of chronic kidney disease among the diabetic population of Nablus, Palestine [11]. This reinforces the critical need for awareness and education on BMI management to prevent diabetes-related complications.

More than half of the participants held at least a bachelor's degree (54.7%), suggesting a relatively educated sample. Although not statistically significant (p=0.077), the highest knowledge scores were observed among those with a master's or PhD (4.93±1.90). This indicates that higher educational levels may be linked to better health literacy. Health literacy is a valuable asset that empowers individuals to make informed health decisions, enhancing their ability to manage and prevent illness and ultimately improving overall health and well-being [22]. These findings emphasize the importance of educational initiatives to foster better health outcomes across populations. However, our findings demonstrated that employment status did not significantly influence the knowledge scores, suggesting that job-related factors alone may not be adequate predictors of health knowledge in this community.

The current study revealed alarming gaps in health maintenance practices, with 84.2% of participants never having been screened for diabetic nephropathy and 68.7% having never checked their kidney function. Additionally, 71.2% of participants reported not engaging in regular physical activity, further compounding their risk for chronic conditions. It is reported that physical activity is associated with improved renal function and reduced risk of diabetic nephropathy, as shown by an increase in the glomerular filtration rate and a decrease in the urinary albumin-creatinine ratio [23]. Similarly, Aldahr and El-Kader conducted a study in Saudi Arabia [24], which found that aerobic exercise significantly improved kidney function, reduced oxidative stress, and lowered inflammatory markers such as IL-6 and TNF-α in patients with diabetic nephropathy. He et al. further supported these findings, demonstrating a positive correlation between physical activity and eGFR levels [25]. The American Diabetes Association recommends annual screening of urinary albumin (spot urine albumin/creatinine ratio) and eGFR in patients who have had type 1 diabetes for at least five years, in all patients with type 2 diabetes beginning at the time of diagnosis, and in all patients who have comorbid hypertension [26].

Our finding highlights that age is significantly associated with attitude. Individuals aged 50 and above typically exhibit more favorable attitudes and heightened involvement in preventive measures. These results are in line with the study conducted in Qatar and Iran [13,27]. This can be ascribed to an increased awareness of health hazards as individuals encounter the long-term consequences of diabetes and associated comorbidities, such as nephropathy [28]. Our study provides an in-depth analysis of demographic and clinical characteristics related to diabetic nephropathy awareness in Hafr Al Batin.

Limitations

However, several limitations must be considered. The cross-sectional design restricts our ability to establish causality. The use of self-reported data for health practices and BMI may introduce reporting bias. Also, the findings might not extend to other regions with differing socioeconomic and health infrastructure characteristics. Furthermore, the inclusion of more detailed behavioral data and controlling for additional variables like family history and healthcare access could enhance the study's depth and applicability. Additionally, the reliance on digital platforms for data collection could exclude segments of the population with less internet access. 

Future recommendations

Future research should aim to implement and evaluate the effectiveness of tailored educational and health promotion programs in increasing awareness and improving health behaviors related to diabetic nephropathy in this region. It will also be important to explore the barriers to accessing healthcare and preventive services, particularly focusing on the underrepresented groups identified in this study.

## Conclusions

The findings from this study provide a comprehensive overview of the demographic characteristics and health behaviors related to diabetic nephropathy among the general population in Hafr Al Batin, Saudi Arabia. Our results show significant disparities in knowledge levels across gender, age groups, and BMI categories. The female participants showed higher knowledge scores than males, and individuals aged 40-49 years demonstrated the highest knowledge levels. Furthermore, our analysis also highlighted a significant deficiency in health maintenance practices related to diabetic nephropathy. A large portion of the population has never been screened, and many are not regularly checking their kidney function. Additionally, our study revealed a concerning lack of physical activity among participants, compounding their risk for diabetic nephropathy. Given the protective benefits of exercise on kidney health, public health campaigns to promote physical activity could significantly mitigate the risks associated with diabetes and its complications.

## Appendices

Considering the survey's observational cross-sectional design, the collected data underwent analysis and presentation utilizing numerical values and percentages. Microsoft Excel (Microsoft Corporation, Redmond, Washington, United States) was utilized as the software tool for managing and analyzing the data.

Questionnaire

Demographic Characteristics

Age

·      Less than 18

·      From 19 to 29

·      From 30 to 39

·      From 40 to 49

·      Above 50

Gender

·      Male

·      Female

Education level

·      Less than high school

·      High school

·      Diploma

·      Bachelor

·      Master

·      PhD

Resident of any region

·      Khobar

·      Dammam

·      Al Qatif

·      Jubail

·      Dhahran

·      Others

Social status

·      Married

·      Single

Amount of unused or expired medication present at home

·      0-5

·      6-10

·      11-15

·      16-20

·      More than 20

Categories of expired and unused medication present in the household

·      Prescribed medicines

·      Non-prescribed medicines

·      Subcutaneous injection

·      Intramuscular injection

·      Intravenous injection

·      Patches

·      Others

General Knowledge

Current medication disposal methods

·      Household trash bins

·      Toilet flush

·      Return to pharmacy

·      Return to hospital

·      Give/donate medications to other people

·      I do not dispose of medications

·      Other methods

Responsible sector to educate the public about safe disposal of medication

·      Hospitals

·      Pharmacies

·      Social media

·      Medical waste companies

Unsafe disposal of medications associated with harmful consequences on public health and the global environment

·      Highly agree

·      Agree

·      Do not know

·      Disagree

·      Strongly disagree

The presence of designated medication disposal locations or medication disposal processes will improve safe medication waste management practices

·      Highly agree

·      Agree

·      Do not know

·      Disagree

·      Strongly disagree

Locating medication disposal facilities via a smartphone application would assist in safe drug disposal practices

·      Highly agree

·      Agree

·      Do not know

·      Disagree

·      Strongly disagree

Medicines manufactured can be used only for a limited period of time after which they should not be used

·      Yes

·      No

·      Do not know

Storage location in the household

·      Locker

·      Refrigerator

·      Bedroom

·      Living room

·      I do not know

·      Medicine cabinet

Do you read medicines disposal instructions?

·      Yes

·      No

·      Not sure

You may use the expired medicine when you need it

·      Yes

·      No

Specific Knowledge

Antibiotic suspensions after opening and dissolution should be used within 1-2 weeks

·      Yes

·      No

·      I don't know

Eye drops/ointments should be used within 28 days after opening

·      Yes

·      No

·      I don't know

Syrups should be used within six months of bottle opening

·      Yes

·      No

·      I don't know

Separately packaged tablets and capsules can be used until the expiry date mentioned on the package

·      Yes

·      No

·      I don't know

Externally applied creams or ointment contained in a tube should be used within three months of opening

·      Yes

·      No

·      I don't know

Attitude

Is the safe disposal of medicines necessary?

·      Yes

·      No

·      Don't know

·      Others

The best method to dispose of medication from the participant's point of view

·      Return to the pharmacy/hospital

·      Through household trash

·      Through toilet flush

·      Others

If you notice drug deposition at the bottom of the bottle, you may add water and shake it before use

·      Yes

·      No

·      Don't know

·      Others

If you forget a drug for a whole day in the car during summer, you should discard it

·      Yes

·      No

·      Don't know

·      Others

If the color or form of medicine has changed, you can continue to use it

·      Yes

·      No

·      Don't know

·      Others

Practice

Verification of expiry date before administering medications

·      Always

·      Often

·      Sometimes

·      Rare

·      Never

Use of any unused/expired prescribed medication for a relative or a friend

·      Always

·      Often

·      Sometimes

·      Rare

·      Never

Are you interested in reading an internal pamphlet or label of the drug before storage to know storage conditions?

·      Yes

·      No

The period after which you check the expiry date of stored medicines

·      At the time of usage

·      Every six months

·      Every three months

·      Monthly

·      Yearly

·      Do not do so
